# Supplementary material for: Bright room temperature single photon source at telecom range in cubic silicon carbide
Source: Nat Commun. 2018 Oct 5;9:4106. doi: 10.1038/s41467-018-06605-3 (PMC6173757; doi:10.1038/s41467-018-06605-3)
Supplement: Supplementary file 1 — Supplementary Information [file 41467_2018_6605_MOESM1_ESM.pdf]

Supplementary Information

Bright room temperature single photons source at telecom range in cubic silicon carbide

	**Sample A**	**Sample B**
Epitaxy layer?	yes	yes
Epi side surface orientation	(111)	(110)
Epi thickness	1 μm	2.7 μm
Doping	No intended doping	No intended doping
N type?	yes	yes
Substrate (thickness)	Si (1000 μm)	Si (725 μm)

Supplementary Table 1: Detailed sample information.

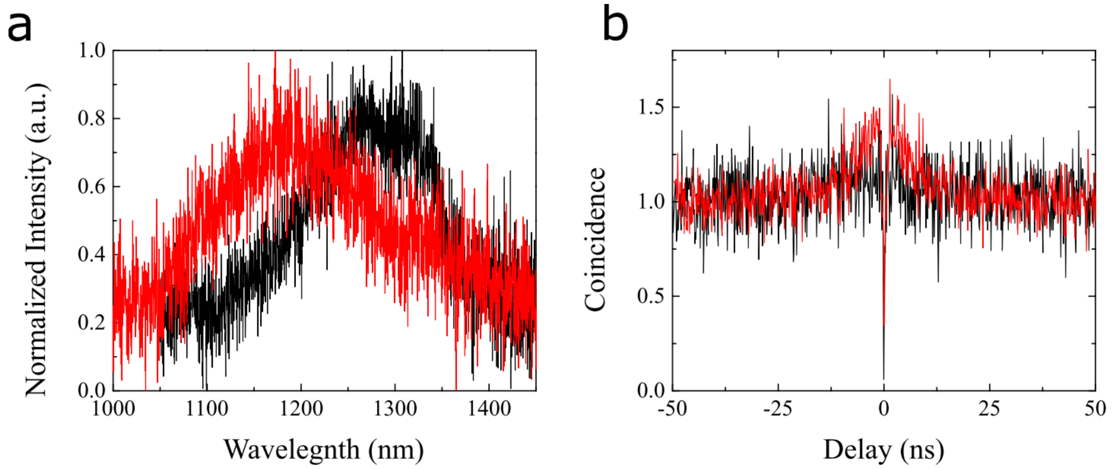

Supplementary Figure 1: SPEs in sample B a. PL of 2 repetitive SPEs in sample B. b. Second-order autocorrelation function of the 2 SPEs to confirm that they are single photon emitters.

Supplementary Note 1: Electron irradiation

We have also irradiated sample A with 1Mev electrons at a range of fluences (E15 cm^{-2} to 5E16 cm^{-2}). After the irradiation, the samples was annealed in 800 degree for 30min in high vacuum. After performing confocal scan, no obvious effect of the electron irradiation (change of SPE density or type) has been observed.

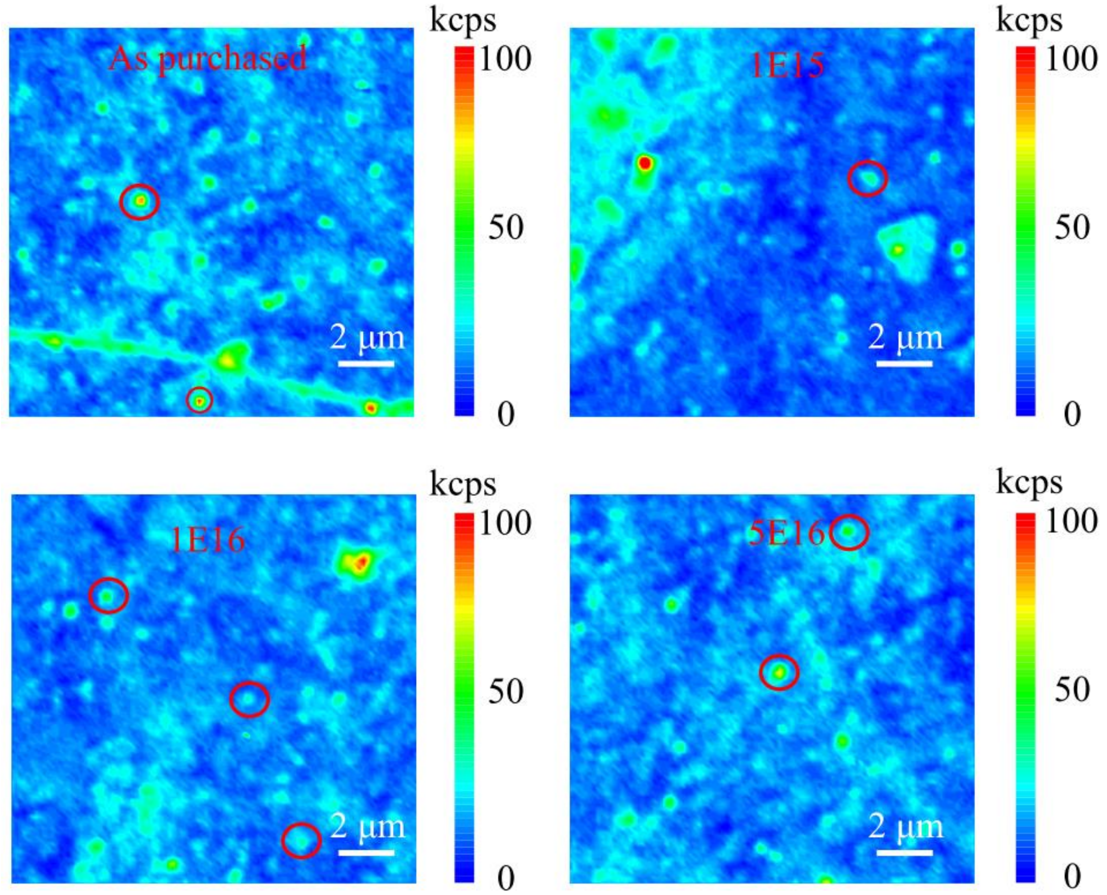

Supplementary Figure 2: Electron irradiation of sample A with difference doses : Confocal map of electron irradiated sample A with different fluences. No clear effect of the irradiation is observed.

Supplementary Note 2: PLE spectra of the SPE

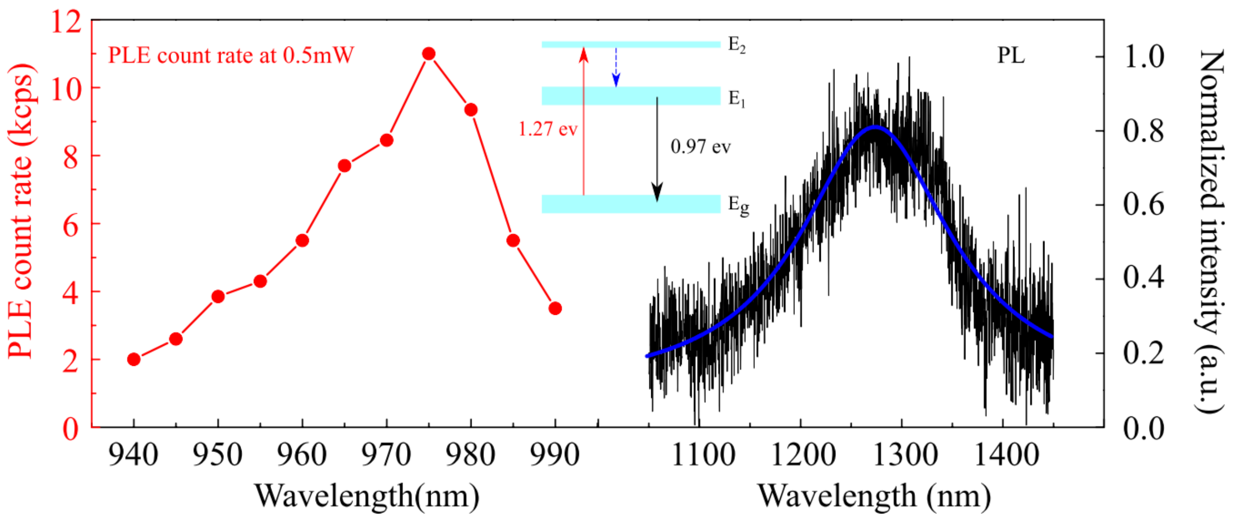

Supplementary Figure 3: PLE spectra of one SPE : The red dots are the raw data of photon counts while tuning the excitation laser wavelength from 940 nm to 990nm while keep the power constant at 0.5 Mw. The black part is the raw data off resonant PL spectrum of the SPE. And the blue solid line is the fitting with Lorenz function. From the fitting, the linewidth is 181 ± 3 nm. Middle figure is the energy level we proposed to explain the PLE spectra.

To know more about the optimal excitation conditions and energy levels of the SPE, we performed PLE excitation measurement. First, we found the SPE which shows a PL spectrum centered at around 1275 nm (0.97 eV) with linewidth 181 ± 3 nm. Then we performed the PLE spectra measurement by tuning the excitation wavelength while keeping the power constant at 0.5mW. The PLE spectra from 940 nm to 990 nm is as shown in red dot in Supplementary Figure 3. According to Franck-Condon principle, maximum position of PLE spectra is blue shifted with

respect to its ZPL position, it is usually asymmetric because of the complex form of the vibronic bands[1]. We found that the most efficient excitation wavelength is around 975 nm (1.27 eV). Based on our observation, we proposed a very simple energy level of the SPE. As displayed in the Supplementary Figure 3, two excited level E_1 ,E_2 and one ground state E_g are involved. The emitter is off resonant excited to E_2 first and then relax to E_1. Then it radiatively goes back to its ground state and emit photons.

[1] Hain, T. C. et al. Excitation and recombination dynamics of vacancy-related spin centers in silicon carbide. J. Appl. Phys. 115, 133508 (2014).
